# The Growth of the Nursing Profession: VII. The Employment of the Waiting Years

**Published:** 1918-04-20

**Authors:** 


### THE MATRONS' AND SISTERS' DEPARTMENT.

### THE GROWTH OF THE NURSING PROFESSION.

### VII. The Employment of the Waiting Years.

-AGE AND TRAINING.

Attention is frequently drawn to the shortness of the working period of a nurse's life, due not only to the strenuousness of the work, but also to the fact that she cannot enter hospital for general training under the age of twenty-one. It has been said by some that this is unreasonable, and that the age limit should be lowered. Nearly all nursing authorities, however, and most doctors, will agree that this would be an unwise procedure for several reasons. The physical strain of a general training is great. Whatever improvements and reforms may be introduced, however the conditions may be ameliorated, the life of a hospital nurse, though extremely healthy, must necessarily be what the outside world calls hard, and is not the sort of life to be undertaken by a girl in her teens. There is also the mental and moral effect to be considered. Here and there one may come across fine natures which develop early, to whom sickness and suffering have appealed for help while they were little more than children, and have brought out the best that was in them. But to plunge the average girl of seventeen or eighteen into the midst of the harrowing sights and sounds of a large hospital must have one of two effects. Either it will, though perhaps unknown to others and having no outward manifestation for years afterwards, exercise an adverse influence on her mental equilibrium and rob her of the gaiety of spirit which is her inalienable birthright, or else she will quickly accustom herself to the sight of suffering and fail to realise what it means. Everyone knows that the best nurses from a practical nursing point of view are those who have been ill themselves. Possibly by reducing the age limit nurses would be turned out more thoroughly equipped with skill and knowledge at an earlier age, but not all the skill and knowledge in the world can avail in the absence of the watchful sympathy which meets a request before it is uttered, and knows when a pillow is uncomfortable or a sound is jarring.

Now, many girls make up their minds to be nurses—though they frequently change them—while they are still quite young. What is to be done with the waiting years? Here is a field which offers great opportunity for initiative, and one would like to see the College of Nursing later on inviting suggestions from its members on this point. Many girls enter for training in children's nursing, which can easily be undertaken at an earlier age. And it might be an idea worthy of consideration and development that every nurse should begin the study of her profession with a year's training in the nursing of children. This would only account for one year, but there are many other ways in which these years might be utilised, and the girl who intends to be a nurse enabled to commence fitting herself for it at a much earlier age than at present. It is sometimes almost pathetic to find how scanty is the education which many good nurses have received. It is not that education alone can make a nurse, for it most emphatically can not, but no good nurse would be the worse off for a more extensive acquaintance with the language and literature of her own and other countries, and for that general development of mental power in its application to life which a liberal education gives. Nor would her lectures present such a baffling problem to her understanding if she already had some knowledge of physics, chemistry, mechanics, and allied subjects.

The nation's nurses are drawn from every class of the community, and the majority of the women who enter the profession have no opportunity of acquiring a liberal education unless they have a natural taste for reading and the patience to inquire and learn for themselves. They frequently, however, possess the intuitive sympathy, genuine kindness, patience, and attention to detail which are a necessary part of a nurse's equipment. It is often heard in hospitals—sometimes as a consolation offered to the despairing—that the best nurses are not those who do well in their exams. Although this is a somewhat sweeping generalisation, there is much truth in it, and we imagine that in a large percentage of the cases in which it is true the underlying cause is a defective or insufficient education. It stands to reason that if education has been limited to the elements of the four R's, with a smattering of history and geography, the pupil-nurse cannot toss about with any real ease or comfort the unfamiliar and high-sounding names of the most ordinary diseases and the simplest drugs, find her way about the complex human organism, or grasp the involutions of the causes and effects of disease. If this fact were clearly realised, we feel sure that something would be done to give the intending nurse facilities not only for a fuller professional education, but also for widening her mental outlook in other directions. It is not enough to provide good books for the nurses to read—they will grow dusty on the shelves so long as the power of appreciation is lacking. In the entrance examination for cadets of the Royal Navy it is not the book-learned boy who gets in; it is the boy who shows himself gifted with that priceless possession known as "gumption." We think the College might do worse than take a leaf out of the Navy's book. If girls of eighteen to twenty who had not reached a fixed standard of education, and who wished to become nurses, were to present themselves for a species of general

☞ Previous articles appeared Jan. 19, 26; Feb. 9, 23; March 16, and April 6, p. 15.

**GROWTH OF NURSING PROFESSION**—(*cont.*).

knowledge examination which would discover to what extent—from the professional standpoint—they were likely to profit by further education, and if the successful candidates were to be given a year or two years' course of general education either at a specially provided institution or by some other method, what would be the result? Would not the pre-eminently nursing qualities which they might possess be strengthened and enriched by the wider outlook thus attained, and the professional spirit be more readily and surely acquired? This may seem at first sight an utterly impracticable idea, but we venture to think that the principle underlying it is sound, and capable of evolving ideas which would be not only workable, but also abundantly fruitful.

